# Nonlinear dynamics of non-equilibrium holes in *p*-type modulation-doped GaInNAs/GaAs quantum wells

**DOI:** 10.1186/1556-276X-6-191

**Published:** 2011-03-02

**Authors:** Hagir Mohammed Khalil, Yun Sun, Naci Balkan, Andreas Amann, Markku Sopanen

**Affiliations:** 1 School of Computer Science and Electronic Engineering, University of Essex, CO4 3SQ, Colchester, UK; 2 Tyndall National Institute, University College Cork, Cork, Ireland; 3 Department of Micro and Nanosciences, Helsinki University of Technology, P.O. Box 3500 FI-02015 TKK, Finland

## Abstract

Nonlinear charge transport parallel to the layers of *p*-modulation-doped GaInNAs/GaAs quantum wells (QWs) is studied both theoretically and experimentally. Experimental results show that at low temperature, *T *= 13 K, the presence of an applied electric field of about 6 kV/cm leads to the heating of the high mobility holes in the GaInNAs QWs, and their real-space transfer (RST) into the low-mobility GaAs barriers. This results in a negative differential mobility and self-generated oscillatory instabilities in the RST regime. We developed an analytical model based upon the coupled nonlinear dynamics of the real-space hole transfer and of the interface potential barrier controlled by space-charge in the doped GaAs layer. Our simulation results predict dc bias-dependent self-generated current oscillations with frequencies in the high microwave range.

## Introduction

During the past decade, dilute nitrides, particularly the quaternary material system of GaInNAs/GaAs, have attracted a great deal of attention, both because of unusual physical properties and potential applications for a variety of optoelectronic devices. The addition of a small amount of nitrogen induces a strong perturbation in the conduction band of matrix semiconductors, while having a negligible effect on the valence band. As a result, the electron mobility is greatly lowered and the hole mobility can become higher than the electron mobility, in materials with relatively high nitrogen content. High hole mobility coupled with the low hole confinement energy (110 meV in our calculation for the samples investigated in this study) [1] in the GaInNAs/GaAs quantum well (QW) structure makes it possible for holes in the well to gain enough energy to overcome the small band discontinuity under an electric field applied parallel to the layer interface, and to transfer into the low-mobility *p*-doped GaAs layer. This leads to a negative differential mobility (NDM) caused by real-space hot hole transfer, as we previously observed [1]. Therefore, under dc conditions, a self-generated current oscillation in the real-space regime, as proposed by Schöll and co-authors [2-5], is expected in *p*-modulation-doped GaInNAs/GaAs heterostructures.

**Figure 1:**
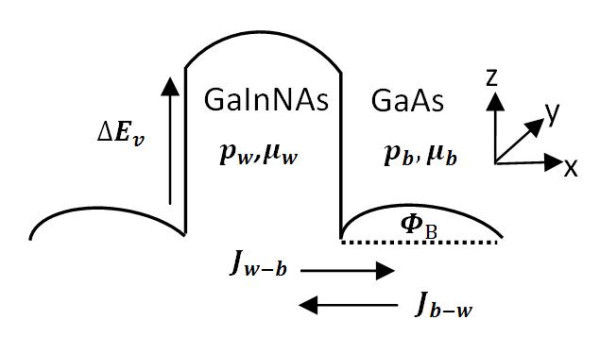
**Schematic energy-band profile of a GaInNAs/GaAs heterostructure**.

In this work, we study the nonlinear charge transport in a modulation-doped GaInNAs/GaAs semiconductor heterostructure where the GaAs barrier layer is intentionally *p*-doped. The charge transport processes perpendicular and parallel to the layers far from thermodynamic equilibrium are modeled by several coupled nonlinear dynamics equations. In this model, self-generated current oscillations can be described in the following way. Real-space transfer (RST) of holes out of the GaInNAs well layer leads to an increase of the hole density in the GaAs barrier, which diminishes the negative space charge that controls the band bending (Figure 1). Consequently, the potential barrier Φ*_B _*decreases, with some delay due to the finite dielectric relaxation time. This leads to an increased thermionic emission backward current *J_b-w _*into the GaInNAs well, which decreases the hole density in the GaAs barrier. As a result, the space charge and Φ*_B _*are increased in the GaAs. This, in turn, decreases the thermionic emission backward current from the well into the barrier [6].

### Negative differential resistance instabilities in *p*-modulation-doped GaInNAs/GaAs QWs

The layer structure of the sample used in this study is given in Table 1. The sample, which was grown by molecular beam epitaxy (MBE) on semi-insulating GaAs substrate, consists of three 7 nm thick GaInNAs QWs, separated by 20 nm thick Be-doped GaAs barriers. These *p*-type-doped barriers are separated from the QWs by 5 nm undoped spacer layers to reduce the remote impurity scattering. The mole fraction of indium and nitrogen in the Ga_1-*x*_In*_x_*N*_y_*As_1-*y *_QWs is *x *= 0.3 and *y *= 0.015, respectively. The sample was fabricated in the shape of a simple bar for *I*-*V *measurement. Fabrication details are given somewhere else [1].

**Table 1:** Numerical parameters used in the simulation for the GaInNAs/GaAs sample [7]

Material	Thickness (Å)	Doping (m^-3^)	
GaAs (cap)	500	Be: 1 × 10^24^	×3
GaAs (barrier)	200	Be: 1 × 10^24^	×3
GaAs (spacer)	50	UD	×3
Ga_1-x_In_x_N_y_As_1-y_ QW	70	UD	×3
GaAs (spacer)	50	UD	×3
GaAs (barrier)	200	Be: 1x10^24^	×3
GaAs (buffer)	500	UD	×3

Semi-insulating GaAs substrate

The nonlinear transport processes depicted in Figure 1 are modeled by a set of dynamic equations relevant to current instabilities in semiconductors. We derive a set of nonlinear partial differential equations for the hole density in the wells (*p_w_*), and in the barriers (*p_b_*), the potential barrier in each of GaAs layers (Φ*_B_*), and the dielectric relaxation of the applied parallel field (*ξ_II_*).

The dynamics of the carrier density in the well and in the barrier are given by [5]

(1) $$\frac{\partial p_{w}}{\partial t}=\frac{1}{qL_{w}}\left(J_{w-b}-J_{b-w}\right)$$

(2) $$\frac{\partial p_{b}}{\partial t}=\frac{1}{qL_{b}}\left(J_{b-w}-J_{w-b}\right)$$

where *J_w-b _*and *J_b-w _*are the thermionic currents flowing from the GaInNAs well layers to the GaAs barrier and from the barrier into the well, respectively, *q *is the positive electron charge, and *L_w _*and *L_b _*are the width of the GaInNAs QW and the GaAs barrier, respectively. The electric field parallel to the layer interface *ξ_II _*can be derived from Poisson's equation and is given by

(3) $$\varepsilon_{0}\varepsilon_{s}\frac{\partial \xi_{II}}{\partial y}=\frac{q}{L_{w}+L_{b}}\left[\left(N_{A}-p_{b}\right)L_{b}+p_{w}L_{w}\right]$$

where *ε*_0 _and *ε_s _*are the absolute and relative permittivity, respectively. Using Equations (1)-(3), the dielectric relaxation of the applied parallel field *ξ_II _*as a function of the current flow (*y*-direction), the transverse space coordinator (*x*-direction), and the time *t *can be written as

(4) $$\begin{array}{l} \varepsilon_{0}\varepsilon_{s}\frac{\partial }{dy}\left(\frac{\partial \xi_{II}}{\partial t}\right)=\frac{q}{L_{w}+L_{b}}\left[\frac{dp_{b}}{dt}L_{b}+\frac{dp_{w}}{dt}L_{w}\right]=\\ \frac{q}{L_{w}+L_{b}}\left[-[\frac{1}{qL_{b}}\left(J_{b-w}-J_{w-b}\right)+\mu_{b}\frac{\partial (p_{b}\xi_{II})}{\partial x}]L_{b}+\left[\frac{1}{qL_{w}}\left(J_{w-b}-J_{b-w}\right)+\mu_{w}\frac{\partial (p_{w}\xi_{II})}{\partial x}\right]L_{w}\right] \end{array}$$

(5) $$=\sigma_{L}\left(\xi_{0}-\xi_{II}\right)-\frac{1}{L_{w}+L_{b}}\left[-qp_{b}\mu_{b}L_{b}\frac{\partial \xi_{II}}{\partial y}\right]+\left[qp_{w}\mu_{w}L_{w}\frac{\partial \xi_{II}}{\partial y}\right]$$

Where *ξ*_0 _= *U*_0_/*d *is the applied field and *U*_0 _is the applied voltage, *σ_L _*= *d*/⌊*h*(*L_w_*+*L_b_*)*qμ_w_R_L_N_A_*⌋ is connected to the load resistance *R_L_*, *d *is the sample length, *h *is the width of the sample, *μ_w _*and *μ_b _*are the hole mobility in the QW and the GaAs barrier, respectively. By integrating both sides of Equation (5), we finally have the dielectric relation of the parallel electric field

(6) $$\frac{\partial \xi_{II}}{\partial t}=J_{II}-\frac{1}{L_{w}+L_{b}}\left[qp_{w}\mu_{w}L_{w}+qp_{b}\mu_{b}L_{b}\right]\xi_{II}$$

where *J_II _*is the external current density flowing through the external circuit at applied bias voltage *U*_0_.

Here, we define the current density flowing through the sample as a function of applied parallel field, using

(7) $$J_{II}=\frac{1}{L_{w}+L_{b}}\left[qp_{w}\mu_{w}L_{w}+qp_{b}\mu_{b}L_{b}\right]\xi_{II}$$

The time-dependent potential barrier in the GaAs layer is given by

(8) $$\frac{\partial \Phi_{B}}{\partial t}=\frac{q^{2}}{\varepsilon_{0}\varepsilon_{s}}\left[-\frac{\mu_{b}N_{A}}{q}\Phi_{B}+\frac{q\mu_{b}L_{b}^{2}}{2\varepsilon_{0}\varepsilon_{s}}{\left(N_{A}-p_{b}\right)}^{2}\right]$$

Equations (1) and (2) represent particle continuity, where the thermionic current densities *J_b-w _*and *J_w-b _*can be calculated using Bethe's theory, by assuming that the width of the space charge is comparable to the mean free path *L_m _*of the holes [4,5]

(9) $$J_{w-b}=-qp_{w}{\left[\frac{K_{B}T_{w}}{2\pi m_{w}^{*}}\right]}^{1/2}e^{\left(\frac{-\Delta E_{v}}{K_{B}T_{w}}\right)}$$

(10) $$J_{w-b}=-qp_{b}{\left[\frac{K_{B}T_{b}}{2\pi m_{b}^{*}}\right]}^{1/2}e^{\left(\frac{-\Phi_{B}}{K_{B}T_{b}}\right)}$$

where $m_{w}^{*}$ and $m_{b}^{*}$ are the hole effective mass in the GaInNAs QW and GaAs barrier, respectively, and the hole temperatures in the well and barrier are approximately given by

(11) $$T_{w}\approx T_{L}+\tau_{E_{w}}q\mu_{w}\xi_{II}^{2}\;T_{b}\approx T_{L}+\tau_{E_{b}}q\mu_{b}\xi_{II}^{2}$$

Since the number of holes in the well and the barrier are related to each other, their total number is conserved [1]:

(12) $$p_{0}L_{w}=p_{w}L_{w}+p_{b}L_{b}$$

where *p*_0 _is the 3 D hole density in the well at low field.

### Numerical results

**Figure 2:**
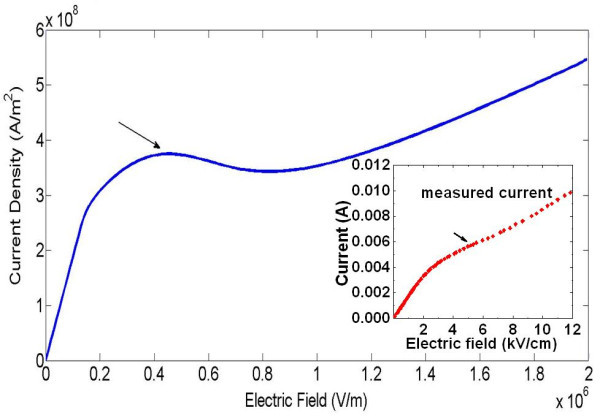
Static current density-field characteristic as a function of the static electric field $\xi_{II}^{*}$. The measured I-V characteristic of p-modulation-doped sample is shown in the inset.

The steady-state can be evaluated by setting Equations (1), (2), (6), and (8) to zero, and using the parameters listed in Table 2. The resulting static current density characteristic as a function of the static electric field is shown in Figure 2. The measured *I*-*V *curve obtained with the same sample in our previous study is placed in the figure inset for comparison [1]. Simulation results predict that the RST of hot holes leads to an N-shaped characteristic with a regime of negative differential resistance [4,7,8]. The critical field for the onset of NDM is the order of 6 kV/cm, which agrees well with our experimental results.

**Table 2:** Numerical parameters used in the simulation for the GaInNAs/GaAs sample [1]

Lw	7 mm
Lb	25 mm
ΔEv	0.12 eV
$m_{\text{w}}^{*}$	0.105 m0
$m_{\text{b}}^{*}$	0.62 m0
d	50 μm
h	28 μm
$\tau_{E_{\text{w}}}$	0.2 ps
$\tau_{E_{\text{b}}}$	0.1 ps
TL	13 K
μw	0.3 m2/Vs
μb	0.021 m2/Vs

**Figure 3:**
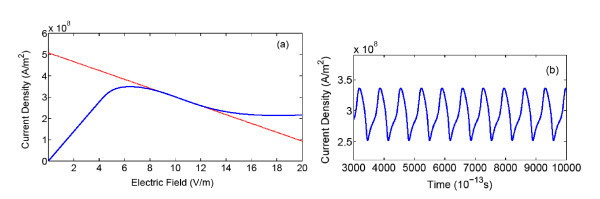
(a) Static current density versus electric field $\xi_{II}^{*}$curve. The load line (straight line) lies within the NDM area to determine the applied dc field. (b) Time-dependent current density curve, with *N_A _*= 2.2 × 10^16^*cm*^-3^

The time-dependent nonlinear Equations (1), (2), (6), and (8) have been numerically resolved using Euler's methods. The simulation reveals that the instability of the dynamic system is strongly dependent on the applied dc bias field, *ξ*_0 _= *U*_0_/*d*. We found that self-generated nonlinear oscillation appears in a range of applied dc electric fields where the load line lies in the NDM regime, as shown in Figure 3a. Figure 3b shows the corresponding current-density oscillations with frequency of 44 GHz, for $\xi_{II}^{*}$ = 10.1 *kV/cm* and *N_A _*= 2.2 × 10^16 ^cm^-3^.

**Figure 4:**
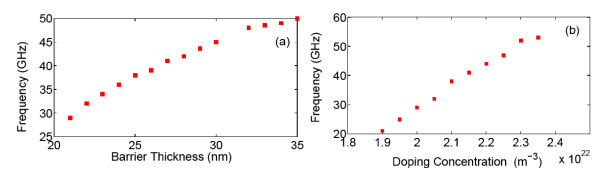
**Oscillation frequency as a function of (a) barrier thickness and (b) doping concentration in the GaAs barrier for *ξ*_0_= 24 kV/cm**.

**Figure 5:**
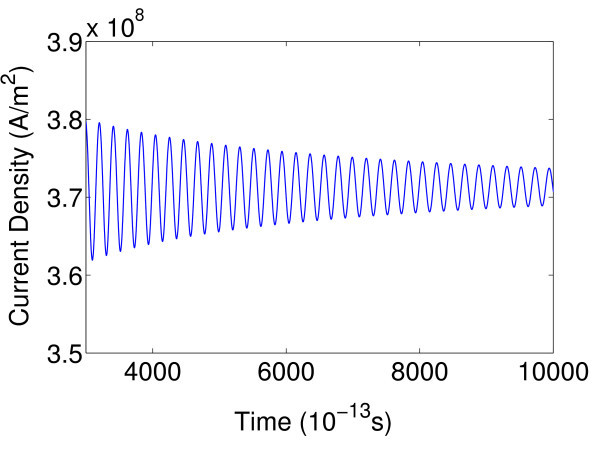
**Periodic oscillation damping with *N_A _*= 2.4 × 10^16^*cm*^-3^**.

It is interesting to find that the oscillation frequency is strongly dependent on the dopant concentration in the barrier and the barrier thickness, as shown in Figure 4. The oscillation frequency increases from 29 to 50 GHz as the dopant concentration in the barrier increases from 1.9 × 10^16 ^*cm*^-3 ^to 2.4 × 10^16 ^*cm*^-3^, accompanied by gradually reduced oscillation amplitude. Finally, the periodic oscillation damps out when the dopant concentration is above 2.4 × 10^16 ^*cm*^-3^, as shown in Figure 5. The oscillation shows similar behavior as the barrier thickness increases. The fact that the self-generated oscillation frequency can be tuned by the doping concentration and the layer width can be explained by the nonlinear combination of the effective thermionic emission time, $\tau_{0}=e^{3/2}L_{\text{w}}\sqrt{3\pi m_{\text{w}}^{*}/\Delta E_{\text{v}}}$ and the dielectric relaxation time, *τ*_r _= ε_0_ε_s_/*qμ*_b_*N*_A _as suggested by Döttling and Schöll [9]. The hysteretic switching transitions between the stable stationary state and the periodic oscillation in a uniform dynamic system depend on the ratio of the effective thermionic emission time and the dielectric relaxation time, γ. In our case, *τ*_0 _= 0.21ps, the change in dopant concentration from 1.8 × 10^16 ^cm^-3 ^to 2.5 × 10^16 ^cm^-3 ^leads to γ increases from 0.076 to 0.12 resulting in phase transition in dynamic system.

## Conclusion

In this work, we studied the transport processes parallel and perpendicular to the layers of *p*-type modulation-doped GaInNAs/GaAs multi-QW structures far from the thermodynamic equilibrium. The simulation results of the steady-state predict an NDM induced by RST of hot holes in the QWs and the critical electric field of the onset of NDM to be the order of 6 kV/cm. This value agrees well with our previous experimental results. The numerically time-dependent simulations indicate that the self-generated oscillation caused by RST with the frequency in the range 20-50 GHz appears under the right applied electric field. The frequency of self-generated oscillation can be flexibly optimized to the range of considerable interest for applications as a simple way of generating high-frequency microwave power based on GaInNAs material system. According to our simulation, the predicted self-generated oscillation can be observed if the GaInNAs QW structure is optimized around 25 nm barrier and less than 2.4 × 10^16 ^cm^-3 ^doping concentration. The current oscillation measurements will be performed using optimized structures fabricated into two terminal devices, and shunted with a 50 Ω resistor and high-speed circuit (high-speed oscilloscope and pulse generator). The experiment results are expected to be published in the near future.

## Abbreviations

NDM: negative differential mobility; QWs: quantum wells; RST: real-space transfer.

## Competing interests

The authors declare that they have no competing interests.

## Authors' contributions

HMK: carried out the theoretical calculations, in collaboration with AA. MS grew the sample according to the specifications. YS fabricated the devices, carried out the experiments. HMK and YS wrote up the article. NB, is the supervisor of the project. All authors read and approved the final manuscript.
